# Supplementary material for: Immunometabolic Profile Associated with Progressive Damage of the Intestinal Mucosa in Adults Screened for Colorectal Cancer: Association with Diet
Source: Int J Mol Sci. 2023 Nov 17;24(22):16451. doi: 10.3390/ijms242216451 (PMC10671025; doi:10.3390/ijms242216451)
Supplement: Supplementary file 1 [file ijms-24-16451-s001.zip › ijms-2695521-supplementary.pdf]

Supplementary Materials

Table S1. Differences in inflammation parameters according to clinical diagnosis groups.

Parameter	Control	Polyps	CRC
Antioxidant capacity (mM)	0.27 ± 0.04 (22)	0.29 ± 0.06 (29)	0.29 ± 0.05 (6)
Total cholesterol (mg/dL)	255.74 ± 40.79 (20)	243.43 ± 72.23 (27)	283.00 ± 87.98 (5)
Cytokines and chemokines			
IL-17 (pg/mL)	13.89 ± 13.14 (23)	17.13 ± 25.55 (33)	21.37 ± 29.32 (6)
IL-6 (pg/mL)	22.40 ± 4.90 (23)	27.08 ± 15.85 (33)	30.14 ± 23.11 (6)
IL-10 (pg/mL)	86.67 ± 24.59 (23)	87.12 ± 20.25 (33)	94.61 ± 37.39 (6)
IL-12 (pg/mL)	42.41 ± 6.43 (23)	44.32 ± 11.80 (33)	53.18 ± 17.82 (6)
IL-4 (pg/mL)	5.08 ± 2.20 (23)	7.42 ± 9.42 (33)	5.39 ± 2.16 (6)
IFN-γ (pg/mL)	12.91 ± 18.64 (23)	8.40 ± 6.67 (33)	15.45 ± 14.27 (6)
TGF-β (pg/mL)	105.84 ± 29.52 (23)	113.86 ± 40.17 (33)	120.23 ± 41.29 (6)
IL-2 (pg/mL)	7.61 ± 7.28 (23)	8.00 ± 7.47 (33)	6.28 ± 1.47 (6)
CXCL10 (pg/mL)	46.15 ± 13.84 (23)	54.20 ± 28.47 (33)	112.82 ± 146.79 * (6)
IL-1β (pg/mL)	30.03 ± 5.40 (23)	39.17 ± 25.47 (33)	36.29 ± 12.96 (6)
TNF-α (pg/mL)	64.63 ± 28.33 (23)	73.33 ± 53.34 (32)	71.06 ± 19.16 (6)
CCL2 (pg/mL)	69.74 ± 49.54 (23)	81.91 ± 90.37 (33)	67.09 ± 17.16 (6)
Adipokines			
Adiponectin (ng/mL)	204376.99 ± 76815.36 (23)	159034.48 ± 63473.78 * (32)	133043.19 ± 36979.07 * (6)
Adipsin (ng/mL)	2384.29 ± 337.36 (23)	2202.65 ± 400.04 (32)	2194.81 ± 278.95 (6)
Leptin (pg/mL)	18.91 ± 3.79 (23)	19.97 ± 7.30 (33)	26.02 ± 12.82 (6)
Resistin (ng/mL)	25.93 ± 20.78 (23)	25.01 ± 19.6 (33)	9.38 ± 7.46 $^{+}$ (6)

Values are shown as mean ± standard deviation (SD) and number of volunteers with available information in parentheses. (*) Statistically significant differences compared to control group ($p < 0.05$). ($^{+}$) Statistically significant differences compared to polyps group ($p < 0.05$). Differences were analyzed by non-parametric tests. CRC, colorectal cancer; IL, interleukin; IFN-γ, interferon-gamma; TGF-β, transforming growth factor-beta; CXCL10, C-X-C motif chemokine ligand 10; TNF-α, tumour necrosis factor-alpha; CCL2, chemokine (C-C motif) ligand 2.

Table S2. Food groups consumption according to clinical diagnosis groups.

Food group (g/day)	Control (*n* = 37)	Polyps (*n* = 49)	CRC (*n* = 3)
Oils and fats	22.81 ± 11.2	26.54 ± 21.52	25.41 ± 13.16
Cereals and cereals products	183.86 ± 124.31	183.69 ± 93.36	176.76 ± 95
Whole grain cereals	26.82 ± 67.53	9.78 ± 26.85	18.21 ± 44.97
Milk and dairy products	338.15 ± 227.7	372.08 ± 230.56	449.65 ± 122.45
Meat and meat products	170.06 ± 101.78	193.44 ± 132.03	172.72 ± 83.1
Eggs	40.54 ± 26.87	46.57 ± 25.18	38.16 ± 24.85
Fish	60.54 ± 43.62	55.53 ± 31.89	69.23 ± 51.88
Seafood	17.41 ± 18.23	17.39 ± 16.79	18.41 ± 17.91
Vegetables	262.07 ± 156.87	253.46 ± 164.55	245.91 ± 156.56
Legumes	35 ± 39.43	32.09 ± 28.59	35.03 ± 38.57
Potatoes and tubers	64.01 ± 54.73	67.2 ± 45.71	80.08 ± 60.78
Fruits	152.17 ± 117.17	137.87 ± 146.59	154.98 ± 41.64
Nuts and seeds	11.81 ± 13.51	9.56 ± 11.13	11.67 ± 12.58
Sugar and sweets	17.07 ± 20.82	15.97 ± 16.22	16.25 ± 14.06
Sauces and condiments	10.13 ± 7.44	11.59 ± 10.79	8.28 ± 2.34
Other foods	22.11 ± 46.74	21.17 ± 44.72	24.86 ± 42.7
Non-alcoholic beverages	1669.98 ± 493.18	1755.13 ± 807.48	1947.74 ± 584.16
Alcoholic beverages	159.2 ± 263.48	375.03 ± 593.13 *	206.18 ± 252.17
Red meat	62.33 ± 56.19	73.15 ± 60.66	68.71 ± 39.37
Processed meat	65.52 ± 50.87	71.19 ± 75.42	61.87 ± 55.3
White meat	45.76 ± 35.42	54.93 ± 37.68	44.68 ± 19.31

Values are shown as mean ± standard deviation (SD) and number of volunteers with available information in parentheses. (*) Statistically significant differences compared to control group ($p < 0.05$). Differences were analyzed by non-parametric tests. CRC, colorectal cancer.

Table S3. Food groups consumption according to aberrant crypt foci (ACF) presence in intestinal mucosa of volunteers diagnosed with intestinal polyps.

Food group (g/day)	ACF presence	
	**No (n = 34)**	**Yes (n = 8)**
Oils and fats	23.68 ± 15.01	30.8 ± 18
Cereals and cereals products	186.65 ± 101.32	165.97 ± 69.58
Whole grain cereals	9.69 ± 26.94	6.23 ± 16.2
Milk and dairy products	392.49 ± 230.51	380.48 ± 271.04
Meat and meat products	171.12 ± 90.45	218.82 ± 133.09
Eggs	44.9 ± 22.64	46.34 ± 29.63
Fish	56.46 ± 34.19	45.6 ± 27.33
Seafood	14.33 ± 14	34.01 ± 23.94 *
Vegetables	261.26 ± 189.27	257.89 ± 104.16
Legumes	28.45 ± 21.13	34.04 ± 25.59
Potatoes and tubers	66.5 ± 45.71	78.17 ± 62.07
Fruits	154.68 ± 152.66	120.58 ± 164.17
Nuts and seeds	10.26 ± 11.2	9.56 ± 13.33
Sugar and sweets	15.94 ± 15.6	14.3 ± 10.88
Sauces and condiments	10.66 ± 7.66	13.33 ± 14.57
Other foods	21.47 ± 52	18.08 ± 24.4
Non-alcoholic beverages	1738.15 ± 859.69	1615.58 ± 527.71
Alcoholic beverages	375.15 ± 663.8	268.72 ± 219.55
Red meat	66.78 ± 57.87	79.14 ± 46.3
Processed meat	56.16 ± 44.53	85.39 ± 89.79
White meat	52.81 ± 36.12	63.43 ± 48.94

Values are shown as mean ± standard deviation (SD) and number of volunteers with available information in parentheses. (*) Statistically significant differences between groups ($p < 0.05$). Differences were analyzed by non-parametric tests. ACF, aberrant crypt foci.

Table S4. Dietary compounds intake according to clinical diagnosis groups.

Compounds	Control (*n* = 37)	Polyps (*n* = 49)	CRC (*n* = 3)
Total protein (g/day)	104.73 ± 36.84	106.74 ± 35.65	115.9 ± 26.42
Animal protein (g/day)	74.63 ± 32.12	76.98 ± 31.61	84.6 ± 21.51
Vegetal protein (g/day)	28.24 ± 11.68	27.87 ± 10.95	28.01 ± 10.35
Total lipids (g/day)	92.1 ± 36.24	97.25 ± 44.31	102.09 ± 27.73
Saturated fatty acids (g/day)	29.95 ± 14.54	31.49 ± 15.48	36.46 ± 11.04
Monounsaturated fatty acids (g/day)	39.47 ± 15.35	41.78 ± 19.47	42.01 ± 14.5
Polyunsaturated fatty acids (g/day)	15.48 ± 6.66	16.2 ± 8.98	15.66 ± 5.35
Total carbohydrates (g/day)	196.01 ± 91.23	197.54 ± 67.96	219.28 ± 72.88
Total dietary fiber (g/day)	22.7 ± 9.36	20.77 ± 7.77	22.71 ± 7.74
Insoluble fiber (g/day)	13.82 ± 7.19	12.49 ± 5.19	13.57 ± 5.38
Soluble fiber (g/day)	2.79 ± 1.43	2.5 ± 1.04	2.69 ± 0.85
Hemicelulose (I) (g/day)	4.63 ± 3.19	3.9 ± 1.81	4.52 ± 2.18
Hemicelulose (S) (g/day)	1.87 ± 1.11	1.72 ± 0.78	1.79 ± 0.74
Cellulose (I) (g/day)	5.47 ± 2.68	5.12 ± 2.06	5.43 ± 2.15
Pectin (I) (g/day)	1.63 ± 0.78	1.48 ± 0.78	1.63 ± 0.46
Pectin (S) (g/day)	0.86 ± 0.57	0.72 ± 0.42	0.83 ± 0.33
Klason lignin (g/day)	1.95 ± 1.18	1.83 ± 0.9	1.84 ± 0.96
Vitamin A (µg R.E/day)	799.69 ± 398.26	777.12 ± 342.67	1088.42 ± 414.49 $^{+}$
Vitamin B1 (mg/day)	1.82 ± 0.66	1.79 ± 0.88	1.79 ± 0.68
Vitamin B12 (µg/day)	7.98 ± 3.83	8.64 ± 3.38	10.35 ± 1.99
Vitamin B2 (mg/day)	1.85 ± 0.68	1.91 ± 0.64	2.06 ± 0.35
Vitamin B6 (mg/day)	2.43 ± 0.77	2.37 ± 0.76	2.43 ± 0.49
Vitamin C (mg/day)	153.1 ± 78.76	158.53 ± 98.62	156.93 ± 57.73
Vitamin D (µg/day)	4.82 ± 2.92	5.07 ± 3.6	5.81 ± 3.15
Vitamin E (mg E.T/day)	11.6 ± 5.06	12.14 ± 6.71	11.96 ± 3.79
Folic acid (µg/day)	338.89 ± 118.29	343.43 ± 135.02	354.06 ± 98.88
Calcium (mg/day)	997.3 ± 451.74	965.26 ± 347.48	1209.98 ± 214.49 $^{+}$
Zinc (mg/day)	11.65 ± 4.61	11.47 ± 3.67	12.52 ± 2.94
Phophorus (mg/day)	1565.99 ± 560.66	1568.49 ± 455.02	1758.32 ± 240.64
Iron (mg/day)	15.19 ± 4.95	16.38 ± 6.17	15.87 ± 3.29
Magnesium (mg/day)	335.35 ± 109.79	333.13 ± 100.75	355.89 ± 55.55
Potassium (mg/day)	3598.93 ± 1019.16	3709.59 ± 1122.11	3891.69 ± 641.04
Selenium (µg/day)	4.1 ± 5.92	3.79 ± 8.08	8.72 ± 15.75
Sodium (mg/day)	3334.65 ± 1404.68	3393.44 ± 1685.51	3623.42 ± 1619.36
Total polyphenols (mg/day)	1578.42 ± 777.42	1615.74 ± 1015.52	1682.65 ± 885.68
Flavonoids (mg/day)	175.21 ± 120.2	286.85 ± 345.35	260.19 ± 219.78
Lignans (mg/day)	63.79 ± 46.72	58.12 ± 70.45	62.99 ± 31.56
Other polyphenols (mg/day)	39.27 ± 43.4	33.22 ± 38.42	40.85 ± 36.41
Phenolic acids (mg/day)	610.82 ± 425.24	499.23 ± 412	634.2 ± 464.98
Stilbenes (mg/day)	0.9 ± 2.43	3.03 ± 6.85	1.91 ± 3.76
ORAC total	5394.79 ± 3520.51	7063.27 ± 7951.55	5862.57 ± 3487.33
ORAC hydrophilic	5246.67 ± 3475.62	6945.9 ± 7928.78	5774.74 ± 3509.21
ORAC lipophilic	155.93 ± 122.86	124.53 ± 100.24	92.44 ± 50.44
Ethanol (g/day)	7.08 ± 10.48	18.72 ± 26.11 *	10.11 ± 11.15
Total PAH (µg/day)	1.08 ± 0.64	1.16 ± 0.49	1.24 ± 0.41
B(a)P (µg/day)	0.06 ± 0.03	0.07 ± 0.03	0.06 ± 0.03
DiB(a)A (µg/day)	0.09 ± 0.18	0.16 ± 0.28 *	0.15 ± 0.24
Total HAs (ng/day)	269.62 ± 441.18	559.94 ± 1568.05	175.42 ± 102.72
PhIP (ng/day)	199.39 ± 318.42	438.41 ± 1253.78	117.5 ± 93.43
DiMeIQx (ng/day)	18.28 ± 40.36	36.6 ± 119.83	15.28 ± 16.58

Compounds	Control (n = 37)	Polyps (n = 49)	CRC (n = 3)
MelQx (ng/day)	50.35 ± 95.57	83.58 ± 209.91	41.65 ± 33.52
MelQ (ng/day)	1.43 ± 1.66	0.9 ± 1.06	0.83 ± 1.12
Nitrates (mg/day)	121.89 ± 103.85	105.13 ± 81.44	111.66 ± 72.04
Nitrites (mg/day)	3.3 ± 2.56	4.65 ± 10.77	3.3 ± 3.04
NDMA (µg/day)	0.2 ± 0.14	0.23 ± 0.22	0.22 ± 0.12
NPIP (µg/day)	0.1 ± 0.08	0.09 ± 0.1	0.09 ± 0.09
NPYR (µg/day)	0.16 ± 0.14	0.14 ± 0.16	0.14 ± 0.14
Acrylamide (µg/day)	16.23 ± 11.44	16.54 ± 10.94	22.81 ± 15.44

Values are shown as mean ± standard deviation (SD) and number of volunteers with available information in parentheses. (*) Statistically significant differences compared to control group ($p < 0.05$). ($^+$) Statistically significant differences compared to polyps group ($p < 0.05$). Differences were analyzed by non-parametric tests. CRC, colorectal cancer. I, insoluble; S, soluble; ORAC, oxygen radical absorbance capacity; Total PAHs, total polycyclic aromatic hydrocarbons; B(a)P, benzo (a) pyrene; DiB(a)A, dibenzo (a) anthracene; Total HA, total heterocyclic amines; PhIP, 2-amino-1-methyl-6-phenylimidazo (4,5,b) pyridine; DiMeIQx, 2-amino-3,4,8 trimethylimidazo (4,5,f) quinoxaline; MeIQx, 2-amino-3,8 dimethylimidazo (4,5,f) quinoxaline; MeIQ, 2-amino-3,4 dimethylimidazo (4,5,f) quinoline; NDMA, N-nitrosodimethylamine; NPIP, N-nitrosopiperidine; NPYR, N-nitrosopyrrolidine.

Table S5. Dietary compounds intake according to aberrant crypt foci (ACF) presence in intestinal mucosa of volunteers diagnosed with intestinal polyps.

Compounds	ACF presence	
	No (n = 34)	Yes (n = 8)
Total protein (g/day)	102.61 ± 30.5	109.61 ± 42.58
Animal protein (g/day)	72.7 ± 24.49	79.89 ± 40.28
Vegetal protein (g/day)	28.11 ± 11.71	27.5 ± 11.48
Total lipids (g/day)	91.97 ± 37.36	100.23 ± 43.22
Saturated fatty acids (g/day)	30.03 ± 13.05	29.67 ± 15.08
Monounsaturated fatty acids (g/day)	39.27 ± 16.94	47.07 ± 21.59
Polyunsaturated fatty acids (g/day)	15.2 ± 7.53	15.5 ± 6.61
Total carbohydrates (g/day)	198.33 ± 73.09	194.07 ± 50.95
Total dietary fiber (g/day)	21.5 ± 8.25	19.96 ± 8.49
Insoluble fiber (g/day)	12.93 ± 5.53	11.5 ± 5.17
Soluble fiber (g/day)	2.62 ± 1.12	2.23 ± 0.97
Hemicelulose (I) (g/day)	4.03 ± 1.94	3.48 ± 1.64
Hemicelulose (S) (g/day)	1.78 ± 0.82	1.53 ± 0.76
Cellulose (I) (g/day)	5.27 ± 2.1	4.86 ± 2.33
Pectin (I) (g/day)	1.57 ± 0.86	1.35 ± 0.63
Pectin (S) (g/day)	0.79 ± 0.46	0.6 ± 0.3
Klason lignin (g/day)	1.89 ± 0.97	1.69 ± 0.67
Vitamin A (µg R.E/day)	808.64 ± 358.28	629.3 ± 240.55
Vitamin B1 (mg/day)	1.68 ± 0.59	1.87 ± 0.93
Vitamin B12 (µg/day)	8.26 ± 3.26	9.74 ± 4.51
Vitamin B2 (mg/day)	1.91 ± 0.58	1.9 ± 0.84
Vitamin B6 (mg/day)	2.32 ± 0.65	2.43 ± 1.04
Vitamin C (mg/day)	164.71 ± 104.99	153.41 ± 110.94
Vitamin D (µg/day)	4.98 ± 3.55	3.61 ± 2.14
Vitamin E (mg E.T/day)	11.52 ± 5.2	11.25 ± 4.29
Folic acid (µg/day)	354.25 ± 144.24	334 ± 144.09
Calcium (mg/day)	1003.22 ± 350.52	889.54 ± 380.46
Zinc (mg/day)	11.2 ± 3.22	12.11 ± 5.12
Phophorus (mg/day)	1549.18 ± 426.31	1563.66 ± 528.49
Iron (mg/day)	15.98 ± 6.36	17.63 ± 6.42
Magnesium (mg/day)	334.62 ± 107.54	333.66 ± 94.47
Potassium (mg/day)	3704.77 ± 1183.21	3832.88 ± 1075.26
Selenium (µg/day)	3.97 ± 9.37	3.86 ± 4.77
Sodium (mg/day)	3119.58 ± 1363.89	3648.36 ± 2233.26
Total polyphenols (mg/day)	1646.85 ± 1114.45	1477.53 ± 878.06
Flavonoids (mg/day)	306.34 ± 361.08	258.43 ± 324.63
Lignans (mg/day)	63.32 ± 82.27	46.57 ± 38.3
Other polyphenols (mg/day)	37.1 ± 44.43	23.2 ± 12.21
Phenolic acids (mg/day)	489 ± 379.15	391.23 ± 379.95
Stilbenes (mg/day)	3.3 ± 7.75	2.76 ± 3.95
ORAC total	7560.47 ± 9068.59	6881.95 ± 4560.86
ORAC hydrophilic	7433.18 ± 9041.56	6757.92 ± 4559.61
ORAC lipophilic	134.19 ± 104.58	130.39 ± 115.42
Ethanol (g/day)	18.42 ± 28.41	14.81 ± 10.86
Total PAH (µg/day)	1.17 ± 0.53	1.12 ± 0.39
B(a)P (µg/day)	0.07 ± 0.02	0.05 ± 0.02
DiB(a)A (µg/day)	0.18 ± 0.31	0.07 ± 0.09
Total HAs (ng/day)	594.48 ± 1840.22	540.1 ± 864.75

Compounds	ACF presence	
	No (n = 34)	Yes (n = 8)
PhlP (ng/day)	456.46 ± 1461.76	467.7 ± 785.14
DiMelQx (ng/day)	43.92 ± 143.38	18.71 ± 23.75
MelQx (ng/day)	92.56 ± 248.2	52.66 ± 60.21
MelQ (ng/day)	0.98 ± 1.15	0.83 ± 0.89
Nitrates (mg/day)	109.05 ± 83.62	126.77 ± 98.05
Nitrites (mg/day)	4.7 ± 12.67	3.44 ± 3.07
NDMA (µg/day)	0.21 ± 0.17	0.23 ± 0.24
NPIP (µg/day)	0.07 ± 0.06	0.1 ± 0.1
NPYR (µg/day)	0.11 ± 0.08	0.16 ± 0.15
Acrylamide (µg/day)	16.85 ± 11.35	20.01 ± 12.33

Values are shown as mean ± standard deviation (SD) and number of volunteers with available information in parentheses. No statistical significant differences were found between groups. Differences were analyzed by non-parametric tests. ACF, aberrant crypt foci. I, insoluble; S, soluble; ORAC, oxygen radical absorbance capacity; Total PAHs, total polycyclic aromatic hydrocarbons; B(a)P, benzo (a) pyrene; DiB(a)A, dibenzo (a) anthracene; Total HA, total heterocyclic amines; PhIP, 2-amino-1-methyl-6-phenylimidazo (4,5,b) pyridine; DiMeIQx, 2-amino-3,4,8 trimethylimidazo (4,5,f) quinoxaline; MeIQx, 2-amino-3,8 dimethylimidazo (4,5,f) quinoxaline; MeIQ, 2-amino-3,4 dimethylimidazo (4,5,f) quinoline; NDMA, N-nitrosodimethylamine; NPIP, N-nitrosopiperidine; NPYR, N-nitrosopyrrolidine.

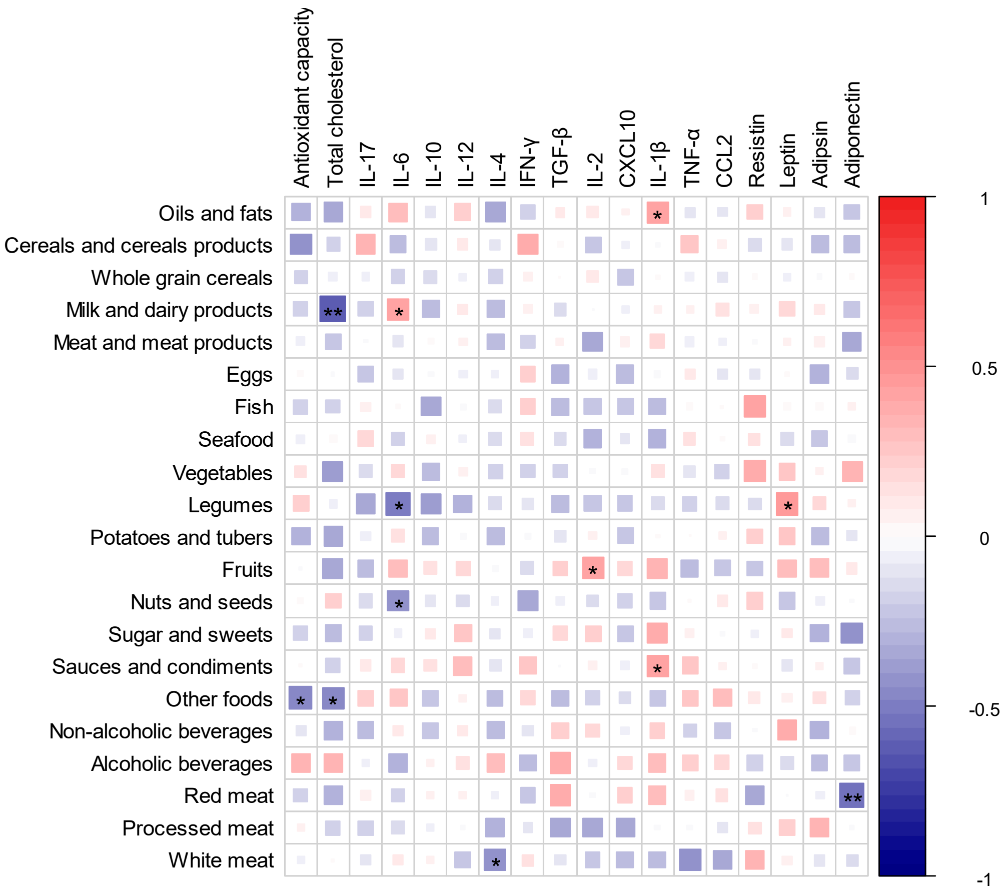

Figure S1. Heatmap defined by Spearman correlations between immunological parameters and food groups in the group of control volunteers. Blue and red colors represent negative and positive associations, respectively. The colour intensity is proportional to the degree of association. (*) $p < 0.05$ (**) $p < 0.001$. IL, interleukin; IFN-γ, interferon-gamma; TGF-β, transforming growth factor-beta; CXCL10, C-X-C motif chemokine ligand 10; TNF-α, tumour necrosis factor-alpha; CCL2, chemokine (C-C motif) ligand 2.

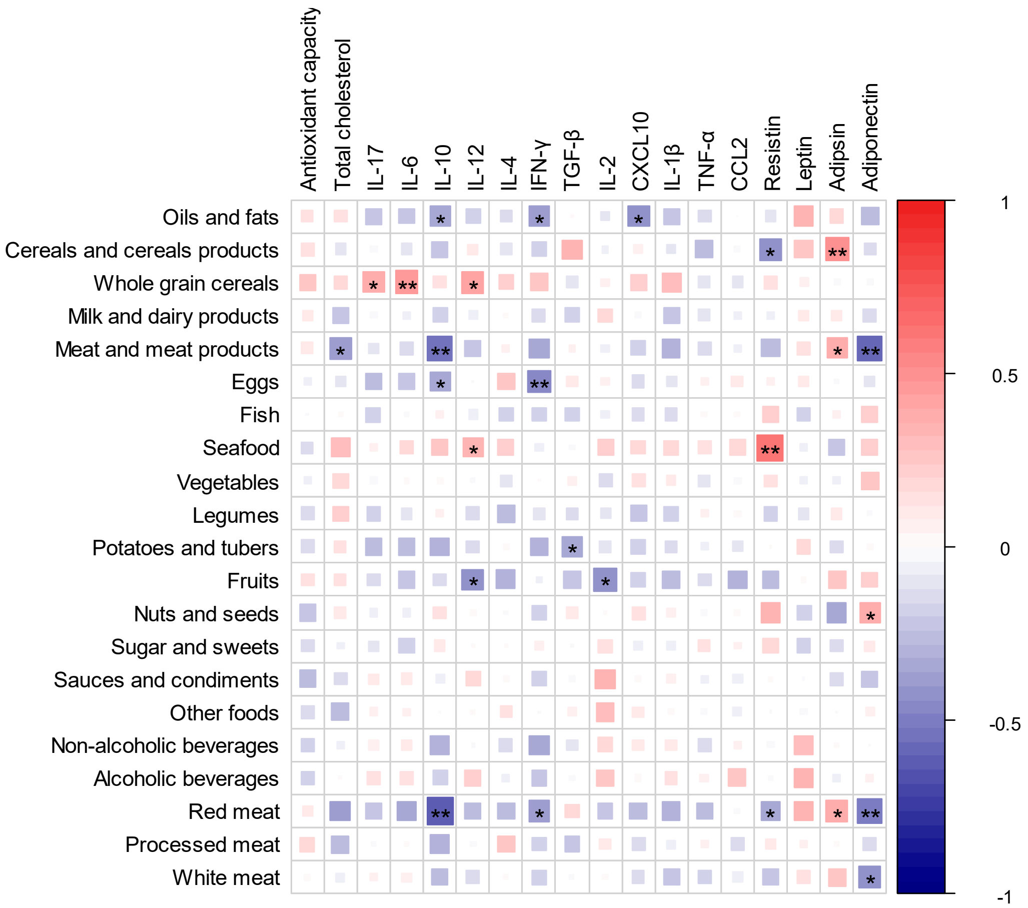

Figure S2. Heatmap defined by Spearman correlations between immunological parameters and food groups in the group of volunteers diagnosed with intestinal polyps. Blue and red colors represent negative and positive associations, respectively. The colour intensity is proportional to the degree of association. (*) $p < 0.05$ (**) $p < 0.001$. IL, interleukin; IFN-γ, interferon-gamma; TGF-β, transforming growth factor-beta; CXCL10, C-X-C motif chemokine ligand 10; TNF-α, tumour necrosis factor-alpha; CCL2, chemokine (C-C motif) ligand 2.

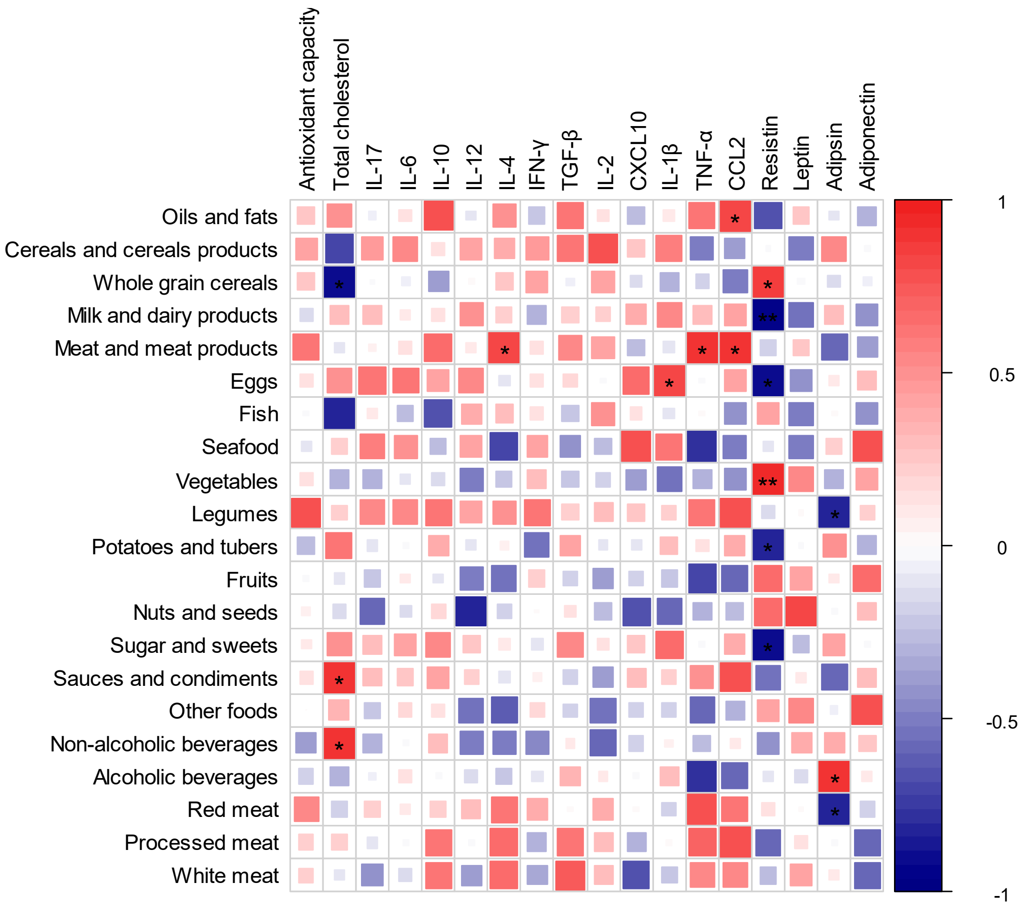

Figure S3. Heatmap defined by Spearman correlations between immunological parameters and food groups in the group of volunteers diagnosed with CRC. Blue and red colors represent negative and positive associations, respectively. The colour intensity is proportional to the degree of association. (*) p < 0.05 (**) p < 0.001. IL, interleukin; IFN-γ, interferon-gamma; TGF-β, transforming growth factor-beta; CXCL10, C-X-C motif chemokine ligand 10; TNF-α, tumour necrosis factor-alpha; CCL2, chemokine (C-C motif) ligand 2.

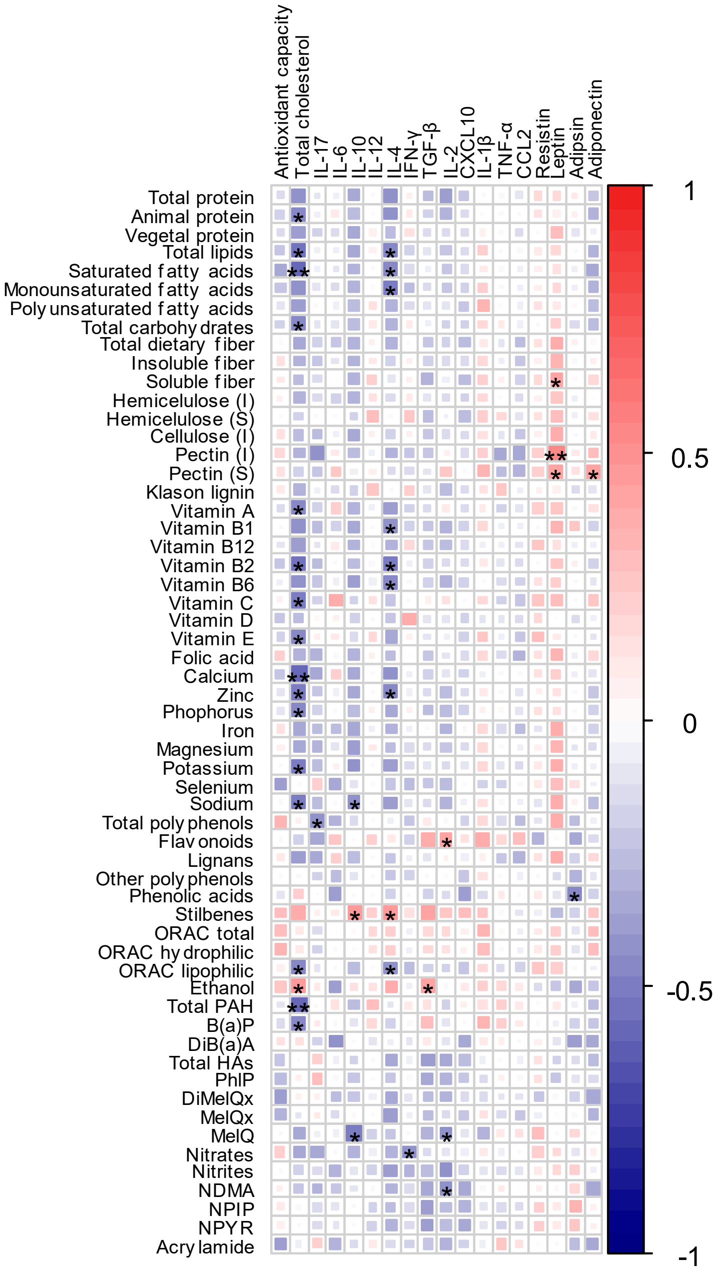

Figure S4. Heatmap defined by Spearman correlations between immunological parameters and dietary compounds in the group of control volunteers. Blue and red colors represent negative and positive associations, respectively. The colour intensity is proportional to the degree of association. (*) $p < 0.05$ (**) $p < 0.001$. IL, interleukin; IFN-γ, interferon-gamma; TGF-β, transforming growth factor-beta; CXCL10, C-X-C motif chemokine ligand 10; TNF-α, tumour necrosis factor-alpha; CCL2, chemokine (C-C motif) ligand 2; I, insoluble; S, soluble; ORAC, oxygen radical absorbance capacity; Total PAHs, total polycyclic aromatic hydrocarbons; B(a)P, benzo (a) pyrene; DiB(a)A, dibenzo (a) anthracene; Total HA, total heterocyclic amines; PhIP, 2-amino-1-methyl-6-phenylimidazo (4,5,b) pyridine; DiMeIQx, 2-amino-3,4,8 trimethylimidazo (4,5,f) quinoxaline; MeIQx, 2-amino-3,8 dimethylimidazo (4,5,f) quinoxaline; MeIQ, 2-amino-3,4 dimethylimidazo (4,5,f) quinoline; NDMA, N-nitrosodimethylamine; NPIP, N-nitrosopiperidine; NPYR, N-nitrosopyrrolidine.

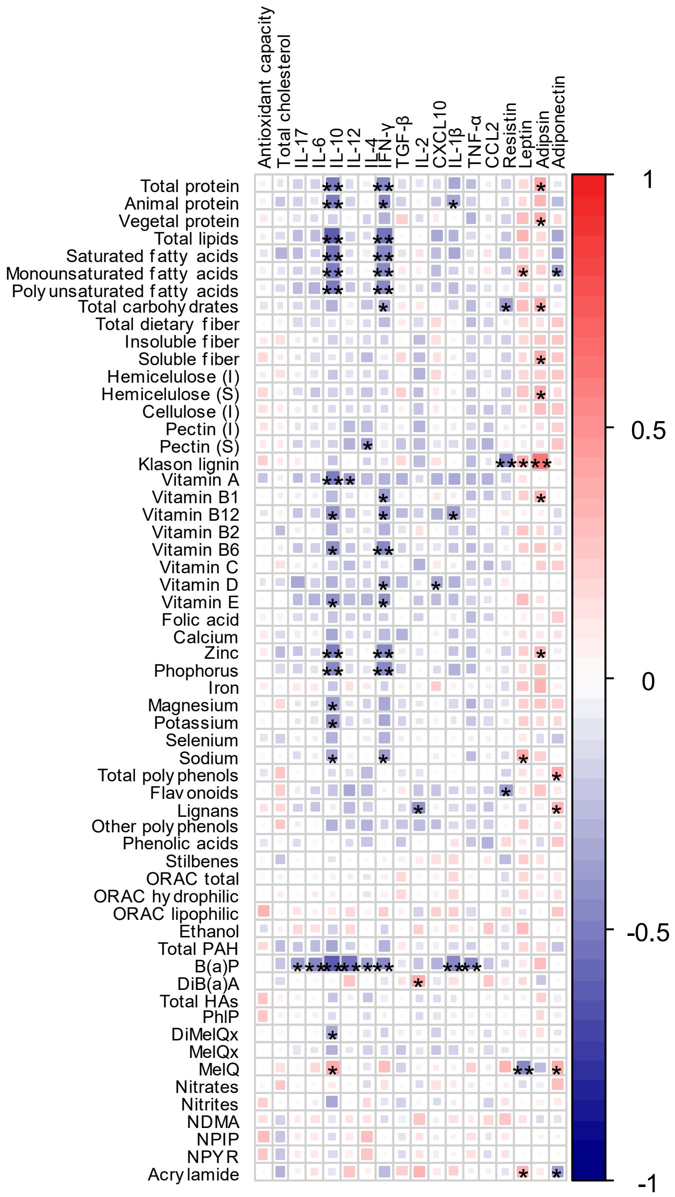

Figure S5. Heatmap defined by Spearman correlations between immunological parameters and dietary compounds in the group of volunteers diagnosed with intestinal polyps. Blue and red colors represent negative and positive associations, respectively. The colour intensity is proportional to the degree of association. (*) $p < 0.05$ (**) $p < 0.001$. IL, interleukin; IFN-γ, interferon-gamma; TGF-β, transforming growth factor-beta; CXCL10, C-X-C motif chemokine ligand 10; TNF-α, tumour necrosis factor-alpha; CCL2, chemokine (C-C motif) ligand 2; I, insoluble; S, soluble; ORAC, oxygen radical absorbance capacity; Total PAHs, total polycyclic aromatic hydrocarbons; B(a)P, benzo (a) pyrene; DiB(a)A, dibenzo (a) anthracene; Total HA, total heterocyclic amines; PhIP, 2-amino-1-methyl-6-phenylimidazo (4,5,b) pyridine; DiMeIQx, 2-amino-3,4,8 trimethylimidazo (4,5,f) quinoxaline; MeIQx, 2-amino-3,8 dimethylimidazo (4,5,f) quinoxaline; MeIQ, 2-amino-3,4 dimethylimidazo (4,5,f) quinoline; NDMA, N-nitrosodimethylamine; NPIP, N-nitrosopiperidine; NPYR, N-nitrosopyrrolidine.

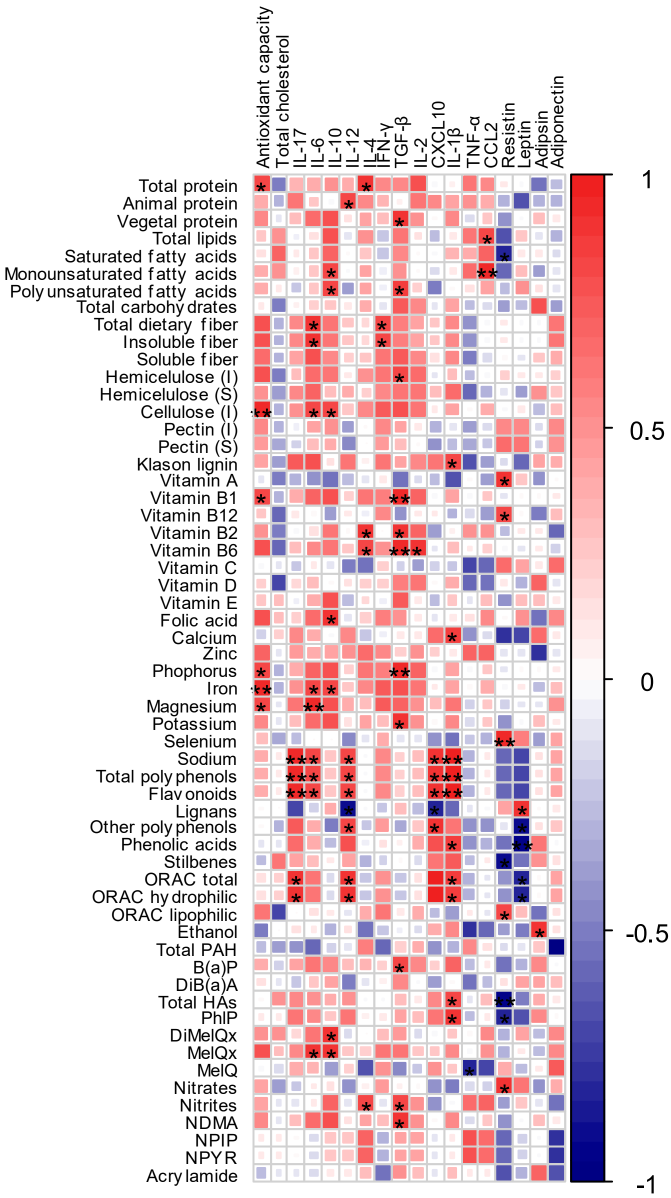

Figure S6. Heatmap defined by Spearman correlations between immunological parameters and dietary compounds in the group of volunteers diagnosed with CRC. Blue and red colors represent negative and positive associations, respectively. The colour intensity is proportional to the degree of association. (*) $p < 0.05$ (**) $p < 0.001$. IL, interleukin; IFN-γ, interferon-gamma; TGF-β, transforming growth factor-beta; CXCL10, C-X-C motif chemokine ligand 10; TNF-α, tumour necrosis factor-alpha; CCL2, chemokine (C-C motif) ligand 2; I, insoluble; S, soluble; ORAC, oxygen radical absorbance capacity; Total PAHs, total polycyclic aromatic hydrocarbons; B(a)P, benzo (a) pyrene; DiB(a)A, dibenzo (a) anthracene; Total HA, total heterocyclic amines; PhIP, 2-amino-1-methyl-6-phenylimidazo (4,5,b) pyridine; DiMeIQx, 2-amino-3,4,8 trimethylimidazo (4,5,f) quinoxaline; MeIQx, 2-amino-3,8 dimethylimidazo (4,5,f) quinoxaline; MeIQ, 2-amino-3,4 dimethylimidazo (4,5,f) quinoline; NDMA, N-nitrosodimethylamine; NPIP, N-nitrosopiperidine; NPYR, N-nitrosopyrrolidine.
